# A Tale of Two Diseases: Decoding Aortic Stenosis and Cardiac Amyloidosis

**DOI:** 10.3390/jcm14082652

**Published:** 2025-04-12

**Authors:** Ioannis Gialamas, George E. Zakynthinos, George Dimeas, Panteleimon Pantelidis, Elias Gialafos, Styliani Brili, Athina Goliopoulou, Ourania Katsarou, Elsi Tryfou, Konstantinos Kalogeras, Gerasimos Siasos, Evangelos Oikonomou

**Affiliations:** 1 3rd Department of Cardiology, “Sotiria” Chest Diseases Hospital, Medical School, National and Kapodistrian University of Athens, 11527 Athens, Greece; jyialamas@gmail.com (I.G.); pan.g.pantelidis@gmail.com (P.P.); gialaf@yahoo.com (E.G.); stlbrili@gmail.com (S.B.); agoliopoulou@gmail.com (A.G.); raniakatsarou@yahoo.gr (O.K.); elsietr@gmail.com (E.T.); kalogerask@yahoo.gr (K.K.); ger_sias@hotmail.com (G.S.); boikono@gmail.com (E.O.); 2 Department of Internal Medicine, General Hospital of Karditsa, 43100 Karditsa, Greece; gedim06@hotmail.com; 3 Cardiovascular Division, Brigham and Women’s Hospital, Harvard Medical School, Boston, MA 02115, USA

**Keywords:** aortic stenosis, cardiac amyloidosis, transthyretin amyloidosis, hereditary transthyretin amyloidosis, low-flow, low-gradient aortic stenosis, wild-type transthyretin amyloidosis, transthyretin, ATTR, AS, cardiomyopathy

## Abstract

**Background/Objectives:** Transthyretin cardiac amyloidosis (ATTR-CA) is an infiltrative cardiomyopathy caused by transthyretin (TTR) amyloid deposition in the myocardium, increasingly recognized in patients with aortic stenosis (AS). This study aims to investigate the diagnostic challenges and therapeutic strategies for patients with both conditions, focusing on shared pathophysiological mechanisms and key diagnostic indicators. **Methods:** A multimodal diagnostic approach was applied, utilizing cardiac magnetic resonance (CMR) and bone scintigraphy with technetium-99m-labeled tracers to assess AS patients with suspected ATTR-CA. Clinical signs, such as disproportionate heart failure symptoms, conduction abnormalities, and low-flow, low-gradient AS, were evaluated. Electrocardiographic findings, including low-voltage QRS complexes and pseudo-infarction patterns, were also assessed. Treatment options, including transcatheter aortic valve replacement (TAVR) and emerging pharmacotherapies for ATTR-CA, were analyzed. **Results:** The study found that ATTR-CA is increasingly prevalent in AS patients, with shared mechanisms like oxidative stress and amyloid-induced tissue remodeling. Key diagnostic signs include disproportionate heart failure symptoms, conduction abnormalities, and specific electrocardiographic patterns. TAVR was effective in both isolated AS and AS with ATTR-CA, although patients with both conditions had a higher risk of heart failure hospitalization and persistent symptoms. Emerging pharmacotherapies, such as TTR stabilizers and gene-silencing agents, showed promise in slowing disease progression. **Conclusions:** A multimodal diagnostic approach is essential for the early detection of ATTR-CA in AS patients. Combining TAVR with emerging pharmacotherapies may improve long-term outcomes for this high-risk group, enhancing patient care in those with both conditions.

## 1. Introduction

There is an increased number of studies showing an increased prevalence of ATTR-CA in patients with aortic stenosis (AS). AS is the most common valvular heart disease among the elderly, affecting over 4% of individuals aged older than 75 years [1]. It is a progressive disease characterized by inflammation, lipid deposition, and the calcification of the aortic valve leaflets, resulting in valve stenosis and, consequently, increased afterload of the left ventricle (LV). AS prevalence rises with age, and it is associated with significant morbidity and mortality if left untreated. Surgical aortic valve replacement (SAVR) has historically been the standard treatment; however, transcatheter aortic valve replacement (TAVR) has emerged as a non-inferior alternative, particularly for elderly or high-risk patients.

Cardiac amyloidosis (CA) is an infiltrative disease caused by the extra-cellular deposition of misfolded amyloid proteins in the myocardium, leading to restrictive cardiomyopathy and heart failure (HF) [2]. The two main subtypes are light-chain (AL-CA) and transthyretin (ATTR-CA) CA amyloidosis, with the latter being further classified into hereditary (hATTR) and wild-type (wtATTR) forms [3]. ATTR-CA, the most common subtype in elderly patients with AS, is increasingly recognized as a significant contributor to HF, particularly in those with disproportionate symptoms despite mild or moderate AS. The prevalence of CA increases with age, reaching up to 25% in octogenarians, according to autopsy studies [4]. Advancements in non-invasive imaging, particularly bone scintigraphy, have enhanced the diagnosis of ATTR-CA, thereby reducing the endomyocardial biopsy [5,6].

The coexistence of ATTR-CA and AS (AS-CA) presents significant diagnostic and therapeutic challenges, given the overlap in epidemiological, clinical, and echocardiographic features [7]. Patients with AS-CA frequently present with right ventricular heart failure (RVHF), conduction abnormalities, low-voltage ECG findings, and disproportionate HF symptoms despite non-severe AS. Emerging pharmacotherapies—such as TTR stabilizers and gene-silencing therapies—have transformed the management of ATTR-CA, while TAVR has demonstrated superior outcomes in AS-CA patients compared to medical therapy alone. Nevertheless, the delayed recognition of CA remains a significant issue in patients with AS, with CA hypertrophy being attributed to AS, hypertensive heart disease, or hypertrophic cardiomyopathy (HCM) [8,9]. Given the increasing awareness of AS-CA and the evolving therapeutic landscape, there is a critical need to standardize diagnostic evaluation and optimize treatment strategies for this high-risk population. In this context, we conducted a narrative review to comprehensively synthesize existing evidence and clinical insights regarding the coexistence of ATTR-CA and AS (AS-CA).

## 2. Pathophysiology of the High Prevalence of Cardiac Amyloidosis in Patients with Aortic Stenosis

CA results from the deposition of normally soluble proteins or peptides as insoluble amyloid fibrils in myocardial tissue. The misfolding of nearly 40 human proteins has been identified as a cause of these fibrils, with five proteins being strongly associated with CA: immunoglobulin light chain, TTR, immunoglobulin heavy chain, serum amyloid A, and apolipoprotein A-I [10]. Among these, TTR misfolding is the most common cause of CA associated with AS, particularly in men over 70 years old [11,12]. As the aging population grows and invasive treatments such as TAVR become more prevalent, studies have increasingly identified cardiac TTR deposition in elderly patients with severe symptomatic AS [13].

Although CA is rare, its prevalence among patients treated for AS has been reported to be approximately 8% [14]. The link between AS and ATTR-CA remains unclear, but several potential mechanisms have been proposed.

### 2.1. Common Epidemiology

One hypothesis suggests that AS and CA share common epidemiologic and age-related features, as both conditions increase in prevalence with age. TTR deposition is frequently observed in elderly individuals (22–25%) at autopsy, though it does not always lead to cardiomyopathy [15,16]. The estimated prevalence of CA in individuals aged ≥ 75 years in the European standard population is 4.15% in males and 1.03% in females, which is lower than the 10% prevalence reported in AS-CA studies [17]. This discrepancy suggests that a purely epidemiological explanation may not fully account for the observed association.

### 2.2. Transthyretin Deposition to Aortic Valve

Another hypothesis proposes that amyloidosis contributes to both cardiomyopathy and AS. Amyloid fibrils can infiltrate cardiovascular structures, including the aortic valve, potentially initiating and accelerating AS [18,19]. Amyloid deposition has been observed within stenotic aortic valve leaflets at a higher prevalence (75%) compared to cardiomyopathy [18,20]. Additionally, these deposits are frequently found near calcified regions, although the specific amyloid proteins responsible for cardiomyopathy remain unidentified [18,21,22,23].

### 2.3. Common Etiology for Both Pathologies

Emerging evidence indicates a shared pathophysiological mechanism between amyloid deposition and aortic valve calcification, with oxidative stress playing a pivotal role in this. In vitro studies demonstrate that oxidation induces amyloid formation in wild-type apolipoprotein A-I and TTR, while acidic pH conditions accelerate both of these outcomes [10,24,25,26].

### 2.4. Aortic Stenosis Is the Etiology of Cardiac Amyloidosis

Furthermore, high shear stress associated with AS has been implicated in promoting amyloidogenic processes and CA development. The mechano-enzymatic hypothesis suggests that AS-related shear stress activates the transition of plasminogen to plasmin, leading to the cleavage of TTR tetramers into amyloidogenic fibrils [27,28]. Increased myocardial strain due to AS may act as a mechanical trigger, promoting amyloid deposition. Notably, reducing shear stress through TAVR may mitigate amyloid fibril aggregation and related inflammatory processes [29]. Additionally, calcium ions from calcified aortic valves may enhance TTR tetramer susceptibility to proteolytic cleavage, further supporting the link between AS and CA [30].

These findings suggest that AS and CA are interconnected through multiple pathophysiological pathways, highlighting the need for further research to clarify their association and optimize diagnostic and therapeutic strategies.

## 3. Clinical Findings Associated with AS and CA

ATTR-CA should be suspected in every case of AS, particularly in elderly male patients with a history of carpal tunnel syndrome (CTS), lumbar spinal stenosis, spontaneous tendon ruptures, premature pacemaker implantation, and disproportionate HF symptoms despite non-severe AS. A prominent indicator is the presence of (RVHF) [31]. Additionally, hearing loss has been reported as a common manifestation [32].

Amyloid deposition in ATTR-CA is not limited to myocardial tissue but can also affect soft tissue structures, including ligaments, leading to conditions such as CTS, lumbar spinal stenosis, and biceps tendon rupture [33]. Among these, CTS is the most frequent in the ATTR-CA population, though its exact pathophysiology remains unclear. While mechanical stress has been suggested as a contributing factor to amyloid deposition, scientific evidence is lacking [33]. An Italian study found CTS history in 14% of hATTR and 25% of wdATTR patients, which is significantly higher than in the general population (4.1%). Notably, CTS was often bilateral and typically preceded a diagnosis of CA by 5 to 9 years. No increased incidence of CTS was observed in patients with AL amyloidosis. Interestingly, in most cases of early-stage wtATTR diagnosis, CTS was an initial presentation [34].

Orthopedic conditions are also prevalent in ATTR-CA. Rubin and colleagues found that 25.9% of wtATTR-CA and 18.8% of hATTR-CA patients underwent hip or knee arthroplasty, with significantly higher surgical rates compared to the general population (hip arthroplasty: RR 5.61, 95% CI: 2.25–4.64; knee arthroplasty: RR 3.32, 95% CI: 2.25–4.64) [33]. A history of multiple orthopedic surgeries should, thus, be considered a red flag for ATTR amyloidosis suspicion [33].

In a cohort study of 411 patients, 289 (70.3%) had CTS, with 74.7% experiencing bilateral CTS preceding CA diagnosis by an average of 9.2 years. The Cochran–Armitage trend test demonstrated a significant association between CTS and systemic manifestations of wtATTR, including lumbar canal stenosis, neuropathy, and hearing loss. Multivariate analysis confirmed that CTS remained independently associated with these conditions, as well as with lower highly sensitive troponin T (hsTnT), increased left ventricular posterior wall thickness, posterior wall hypertrophy, and a higher heart-to-mediastinum ratio. However, no more significant associations were found between CTS and AS, N-terminal pro-B-type natriuretic peptide (NT-proBNP), severe interventricular septal hypertrophy, or amyloid deposits in salivary gland biopsy, though a trend remained for the latter two [34].

Patients with CTS exhibited severe AS exclusively in the low-flow, low-gradient AS (LFLG-AS) phenotype, with a lower proportion of severe aortic valve calcium scores (AVCS) compared to those without CTS. Moreover, severe AVCS patients had significantly lower rates of CTS than those with mild-to-moderate AVCS. This strong and independent association of CTS with extracardiac manifestations suggests a systemic phenotype of wtATTR-CA, whereas patients without CTS appear to have a more cardiac-localized disease [34].

ATTR-CA is more common in males, with prevalence increasing with age. Macroglossia, a hallmark of AL amyloidosis, is notably less frequent in ATTR-CA [35]. Compared to isolated AS, amyloidosis correlates with a worse New York Heart Association (NYHA) functional class (III/IV in 78% vs. 59%, *p* = 0.1), more frequent atrial arrhythmias (67% vs. 20%, *p* = 0.006), and a higher Society of Thoracic Surgery (STS) risk score (6.9% vs. 3.8%, *p* = 0.024) [12,35].

Clinically, ATTR-CA patients usually present with HF with preserved ejection fraction (HFpEF), driven by high filling pressures and low cardiac output, with RVHF being a common feature [36]. Another characteristic is the so-called “natural cure” of hypertension, where patients require down-titration or the discontinuation of antihypertensive medications due to underlying autonomic dysfunction. Additionally, unexplained peripheral or autonomic neuropathy raises the suspicion of hATTR, though it can also be seen in AL-CA and occasionally in wtATTR [37].

Regarding functional status, in a study evaluating isolated AS patients, 55 were categorized as NYHA class II, 36 as NYHA class III, and two as NYHA class IV. Among patients with a positive bone scintigraphy (BS) scan, all were classified as NYHA class III. The positive BS cohort exhibited a higher prevalence of assisted hearing impairment (71% vs. 31%; *p* = 0.07) and trends toward older age (median 86 vs. 82 years), a history of HF (71% vs. 45%), atrial fibrillation (43% vs. 28%), and prior CTS surgery (29% vs. 8%). Although NT-proBNP and hsTnT levels did not reach statistical significance in this cohort, the median RAISE (Remodeling, Age, Injury, System, and Electrical) score, which is a predictor of ATTR-CM risk, was significantly higher in the positive BS group. After Holm–Bonferroni correction, only high rates of pacemaker implantation before TAVR remained statistically significant [38].

A thorough assessment of symptoms is critical, as HF symptoms that seem disproportionate to AS severity may indicate concurrent CA. This is particularly relevant when signs of RVHF, such as peripheral and splanchnic congestion, are prominent [36].

## 4. Electrocardiographic Findings in Cardiac Amyloidosis and Aortic Stenosis

Patients with CA often present with characteristic electrocardiographic (ECG) abnormalities. Low voltages, long considered pathognomonic of CA, occur in nearly 50% of patients with AL CA and approximately 30% of patients with ATTR-CA [36]. Despite this, the low sensitivity of low voltage criteria means they should not be used to rule out the disease, particularly in the context of AS, where left ventricular hypertrophy is expected. Instead, attention should be paid to disproportionate wall thickness measurements relative to the ECG voltage, as CA leads to pseudo-hypertrophy (wall thickening without myocyte mass increase) [36].

Two hallmark ECG findings suggestive of CA include pseudo-infarction patterns (Q waves without a history of myocardial infarction, conduction abnormalities, and atrial arrhythmias [31]—particularly in anterior leads) and low-voltage QRS complexes. The pseudo-infarction pattern is common, occurring in approximately 60% of patients with ATTR-CA. In contrast, the low voltage pattern is observed in only 25–40% of cases, and 7–10% of patients with ATTR-CA may even exhibit LVH on ECG [37].

Patients with concomitant ATTR-CA and AS tend to exhibit more pronounced ECG abnormalities than those with AS alone. Specific electrocardiographic parameters significantly associated with AS and CA include the following:

- A lower Sokolow–Lyon index;
- A longer QRS duration;
- A higher prevalence of right bundle branch block (RBBB) [39].

A study by Castano et al. found that patients with AS and ATTR-CA had a significantly longer QRS duration (127 ms vs. 110 ms) and a higher prevalence of RBBB (37.5% vs. 15.8%) compared to those with isolated AS. The most common arrhythmia in this population was AF, observed in almost half of the patients [12,37]. As amyloid deposition progresses, the direct involvement of the sinoatrial node, atrioventricular node, and bundle branches leads to various conduction abnormalities and arrhythmias, particularly atrial fibrillation, which becomes more frequent with increasing disease severity [37].

Another important ECG clue in CA is the discordance between QRS voltage and LVH on imaging. This discrepancy helps differentiate CA from hypertensive or hypertrophic cardiomyopathy [40]. Additionally, the Sokolow–Lyon index and the voltage/mass ratio (VMR) can aid in diagnosing CA in AS patients. VMR, which combines LV mass index and ECG hypertrophy signs, is typically lower in CA-AS patients, effectively distinguishing them from those with isolated AS. However, caution should be exercised in cases of bundle branch block or pacemaker-induced rhythms, which can affect voltage quantification [37].

Patients with ATTR-CA rarely have a normal ECG. The presence of broader QRS complexes and a higher prevalence of RBBB have been identified as strong predictors of AS-CA [5,41,42]. In one study, patients with a positive BS had a significantly higher male predominance (86% vs. 58%) and an increased history of pacemaker implantation before TAVR (71% vs. 10%) [38].

ECG abnormalities tend to worsen as amyloid deposition increases. In a cohort of 425 ATTR-CA patients, low ECG voltage, pathological Q waves, and prolonged PR, QRS, and QT intervals became more prevalent with increasing interventricular septal thickness and were most common in patients with the thickest hearts. Compared to those with isolated AS, patients with concomitant ATTR-CA and AS exhibited more pronounced ECG abnormalities [7].

## 5. Biomarkers in ATTR-CA and Aortic Stenosis

Laboratory abnormalities indicative of CA include persistently elevated hsTnT levels and disproportionately high NT-proBNP concentrations relative to the degree of LV dysfunction [31,43]. Several studies have shown that NT-proBNP levels are significantly higher in patients with both ATTR-CA and AS than in those with AS alone [42,44]. This is similar to those found in ATTR-CM [45]. HsTnT levels are also higher in AS-ATTR (compared to AS alone (24 ng/L, IQR: 15–39; *p* < 0.001) [5].

## 6. Scoring Systems for Identifying Cardiac Amyloidosis in Aortic Stenosis

The identification of ATTR-CA in patients with AS remains challenging. Although technetium−99m 3,3-diphosphono−1,2-propanodicarboxylic acid (DPD) BS is a highly specific and sensitive diagnostic tool, the systematic screening of all AS patients referred for TAVR is not feasible due to economic and logistical constraints. As a result, clinical scoring systems have been developed to help stratify patients at risk for concomitant CA and guide further diagnostic testing.

### 6.1. The RAISE Score

One such tool is the RAISE score, which is a clinical scoring system designed to screen patients with severe AS for co-existing CA. This score incorporates the following five key domains:

- Cardiac remodeling (LVH and diastolic dysfunction);
- Age;
- Cardiac injury (hsTnT);
- Systemic disease (CTS);
- Electrical disturbances (RBBB and low voltage electrocardiogram).

Each parameter is assigned a distinct score if present, and a total score of ≥2 points should prompt further evaluation with DPD BS and light chain assessment. The scoring system is assigned as follows:

- One point for age ≥ 85 years, Sokolow/Lyon index < 1.9 mV, hsTnT > 20 ng/mL, and E/A ratio > 1.4;
- Two points for the presence of RBBB;
- Three points for a history of CTS.

A score of ≥2 demonstrated a high sensitivity (84%), while a score of ≥3 showed a high specificity (94%) for detecting CA. Among the six parameters, CTS and low electrocardiogram voltage despite LVH were the most specific indicators of co-existing CA [5,46,47].

### 6.2. Electronic Medical Record-Based Scoring System

Looking ahead to 2025, efforts are being made to integrate a scoring logic within electronic medical record (EMR) systems to identify high-risk individuals based on diagnostic patterns. The EMR-based scoring system aims to perform “pattern identification” to flag patients likely to have ATTR-CA. These individuals could then be referred for further screening during routine clinic visits or undergo targeted strain analysis during echocardiography.

The EMR scoring system was developed based on the strength and specificity of associations with ATTR-CA found in the literature. A meta-analysis conducted by our group on the prevalence of ATTR-CA in AS patients found that one in nine AS patients had ATTR-CA. Furthermore, patients with both ATTR-CA and a history of TAVR or SAVR had a significantly higher prevalence of CTS (17% vs. 2%, odds ratio 8). Given the high prevalence of AS and TAVR/SAVR and the specificity of carpal tunnel syndrome, this condition was assigned to have the highest score of five.

Other well-established but less specific associations were assigned lower scores as follows:

- Four points for AF (prevalence 15–40%), atrioventricular block, peripheral neuropathy, RVHF, or pulmonary hypertension;
- Three points for bundle branch block (odds ratio 3);
- Two points for less specific findings, such as hip and knee arthroplasty.

Additional considerations included ordering a CMR in elderly patients with diastolic HF, which may indicate a high clinical suspicion but has up to 20% false-negative results. Similarly, pacemaker implantation is a recognized risk factor for CA but lacks specificity. Laboratory biomarkers such as NT-proBNP and hsTnT—both commonly elevated in CA—were also integrated into the scoring system.

**Figure 1:**
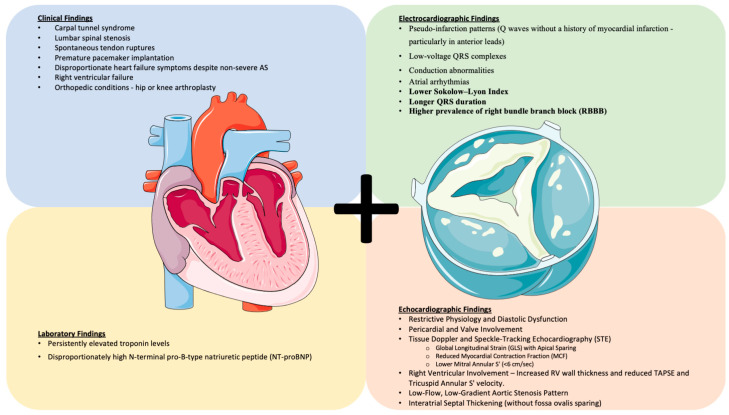
This image presents the echocardiographic, ECG, clinical, and laboratory findings that suggest concomitant amyloidosis in a patient with aortic stenosis. It visually integrates key diagnostic features, highlighting the structural, electrical, and biochemical abnormalities that may indicate myocardial infiltration. (Parts of the figure were drawn using pictures from Servier Medical Art. Servier Medical Art by Servier is licensed under a Creative Commons Attribution 4.0 Unported License (https://creativecommons.org/licenses/by/4.0/, accessed on 12 February 2025).

Patients with an EMR-based high-risk score (≥30) were significantly more likely to exhibit CA-associated echocardiographic findings [48]. (Figure 1).

## 7. The Role of Imaging in the Diagnosis of Aortic Stenosis and Cardiac Amyloidosis

Both AS and CA are characterized by structural and functional abnormalities of the heart, making imaging essential at all stages—from detection and evaluation to confirmation and follow-up. However, differentiating CA from AS poses significant challenges due to their shared characteristics.

Both conditions exhibit LVH with concentric remodeling, impaired systolic performance of the LV (not adequately represented by LVEF), diastolic dysfunction, and elevated LV end-diastolic pressure [49,50]. Given these overlapping features, distinguishing CA when AS is present remains clinically challenging.

### 7.1. The Role of Echocardiography

Echocardiography, particularly transthoracic echocardiography (TTE), is the most widely used imaging technique for cardiovascular diseases due to its availability and feasibility. It provides a comprehensive structural, functional, and hemodynamic assessment, making it the primary modality for detecting heart disease.

However, while TTE is sufficient to establish a diagnosis of AS, this is not the case for CA [51].

In AS, the diagnosis can be made based on echocardiography findings such as the following: (a) the morphological assessment of the aortic valve and measurement of the aortic valve area; (b) evidence of an accelerated transvalvular flow exceeding 4 m/s; (c) Doppler calculation of a mean transaortic valvular gradient > 40 mmHg, and (d) the estimation of an aortic valve area < 1 cm^2^. Pseudo-severe aortic stenosis should be ruled out in cases of low-flow, low-gradient AS using a dobutamine stress test. In paradoxical low-flow, low-gradient AS, echocardiographic diagnosis should be confirmed by cardiac computed tomography (CCT) to establish the presence of significant valvular calcification, indicating true severe stenosis [51].

On the other hand, the diagnosis of CA is based on diagnostic algorithms, with echocardiography serving only as the initial step to raise suspicion or indicate the need for further diagnostic modalities and evaluation. However, echocardiography plays a fundamental role, as most patients undergo further diagnostic testing for CA based on echocardiographic clues. Nevertheless, the characteristic findings suggestive of CA may be masked or overlooked when AS coexists, making differentiation between the two conditions particularly challenging.

#### 7.1.1. Key Echocardiographic Finding in Cardiac Amyloidosis

One of the cardinal echocardiographic features of CA is the increased LV wall thickness, typically ≥ 12 mm, in the absence of hypertension or aortic stenosis. CA is a restrictive cardiomyopathy, and diastolic dysfunction is one of its earliest echocardiographic manifestations. The transmitral Doppler filling pattern typically shows restrictive physiology, characterized by a high early (E-wave)-to-late (A-wave) ratio and an increased E/e′ ratio, reflecting elevated LV filling pressures. In advanced disease, left atrial enlargement, spontaneous echo contrast, and interatrial septal thickening may also be observed [50].

#### 7.1.2. Pericardial and Valve Involvement

Amyloid deposition is not confined to the ventricular myocardium; it also affects the valves and pericardium. As a result, patients with CA frequently exhibit thickened cardiac valves and the presence of pericardial and pleural effusions. Though pericardial effusions are non-specific, they are seen in more than 50% of CA patients, making them a useful ancillary echocardiographic feature [52].

#### 7.1.3. Tissue Doppler and Speckle-Tracking Echocardiography

**Figure 2:**
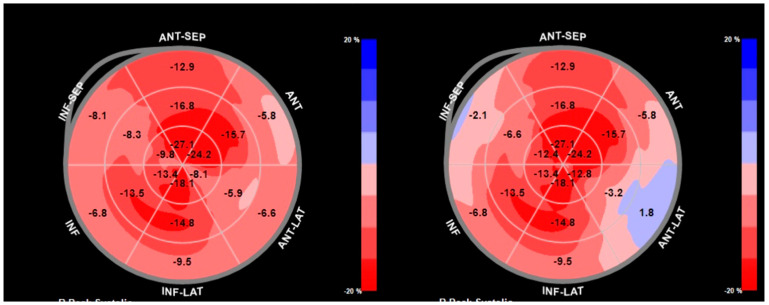
The image depicts two longitudinal strain bull’s−eye plots illustrating the myocardial strain patterns in a patient with cardiac amyloidosis. The plots represent the regional myocardial strain across the left ventricle (LV), with the center indicating the inner portion of the LV and the outer ring representing the outermost myocardial layers. In the plots, the color−coded segments highlight areas of reduced longitudinal strain, particularly in the basal and mid-segments of the LV, which is a common feature in cardiac amyloidosis. The reduced strain values, visible in certain regions, reflect the impaired contractile function due to amyloid deposits that cause myocardial stiffening.

A pivotal advance in echocardiographic assessments is the use of speckle-tracking echocardiography (STE) and tissue Doppler imaging (TDI) to assess longitudinal systolic function. Despite preserved or mildly reduced LVEF, CA is associated with a marked reduction in longitudinal strains. A defining feature is the “apical sparing” pattern of the global longitudinal strain (GLS), also known as the “bull’s-eye” sign, where relative preservation of strain at the apex contrasts with significantly reduced strain in the basal and mid-LV segments. This pattern is highly sensitive (93%) and specific (82%) to CA, helping to differentiate it from other causes of LV thickening, such as hypertensive cardiomyopathy [52]. (Figure 2).

#### 7.1.4. Echocardiographic Indicators of Cardiac Amyloidosis in Coexisting Aortic Stenosis

Several studies have delved into this issue. A recent metanalysis concluded that patients with AS-CA present higher values associated with LV hypertrophy and restrictive physiology, such as interventricular septal thickness, posterior wall thickness, relative wall thickness, the LV mass index, E/A ratio, and LA dimension compared to those with AS only. Conversely, markers of systolic function, including myocardial contraction fraction, average mitral annular S′, tricuspid annular plane systolic excursion (TAPSE), and tricuspid annular S′, were significantly lower in AS-CA patients, indicating a greater impairment in both LV and right ventricular systolic performance. However, based on the existing literature, a cut-off to reliably differentiate between AS or the coexistence of AS and CA cannot be established [53].

In most cases, AS without coronary artery disease does not present impaired LVEF. Therefore, the presence of reduced LVEF in AS should raise suspicion for the coexistence of CA [54]. Indeed, as LV systolic performance declines over time in both aortic stenosis and CA, LVEF remains preserved due to compensatory mechanisms, including LV hypertrophy and cavity remodeling, and notably, a reduction in LV end-diastolic volume [55]. Therefore, in both conditions, impaired LV systolic performance can be assessed using the stroke volume index [55]. The ratio of LV stroke volume, measured via Doppler in the left ventricular outflow (LVOT), to myocardial volume—expressed as the myocardial contraction fraction (MCF)—is reduced in both AS and CA. Notably, even lower values have been observed when both conditions coexist [53].

Average mitral annular S′ has also been evaluated as a measure of LV systolic performance beyond LVEF, with a cut-off value of less than 6 cm/sec proposed as a valuable tool for identifying the coexistence of AS and CA [56].

The LV strain, particularly GLS, is an important marker of LV systolic performance. Any condition associated with impaired LV systolic function, including both AS and CA, can present with reduced LV-GLS values. However, regional LV strain analysis is crucial for differentiation, as CA is characterized by preserved strains in apical segments compared to basal LV segments, resulting in the distinctive “apical sparing” pattern. This phenomenon occurs due to the lower extracellular deposition of amyloid fibrils in the apical region. When apical sparing is preserved, it serves as a highly sensitive and specific tool for distinguishing LVH due to CA from other causes of LVH [57].

However, apical sparing is also commonly observed in patients with AS, likely due to the hemodynamic stress exerted in proximity to the stenotic aortic valve. As a result, this characteristic LV strain pattern may not be sufficient to reliably differentiate between isolated AS and coexisting AS and CA [53].

Interestingly, studies consistently support the fact that most patients with CA and AS present with the low-flow, low-gradient aortic stenosis pattern, and accordingly, this diagnosis may be used as an additive red flag to raise the suspicion of concomitant CA with AS [58,59].

In the context of diastolic function, both AS and CA are associated with severe diastolic dysfunction, characterized by impaired LV relaxation and elevated filling pressures. However, when AS and CA coexist, LV relaxation deteriorates even further [53].

In CA, beyond the LVH and fibrosis seen in AS, amyloid deposition within the LV exacerbates the abnormal filling pattern, often resulting in a restrictive filling pattern, which is a hallmark of CA [6]. Additionally, the amyloid infiltration of the atria may further impair atrial mechanical function, leading to reduced atrial forward forces [60].

A distinguishing feature of CA is the infiltration of the interatrial septum, which notably does not spare the fossa ovalis. This characteristic helps differentiate CA from other conditions, such as lipomatous infiltration [61].

LA strain analysis is a useful tool for assessing atrial function, including its expansion during ventricular systole (reservoir function), early diastolic emptying (conduit function), and contraction. In cardiac CA, studies indicate impaired atrial systolic contraction and a decrease in reservoir function, resulting in an atrium that primarily serves as a conduit throughout the cardiac cycle—often referred to as restrictive atrial dysfunction. These strain parameters play a crucial role in differentiating CA from other HCMs. Notably, ATTR-CA is often associated with more pronounced reductions in the LA reservoir function than AL-CA [62].

One of the key structural indicators distinguishing isolated AS from ASCA is the presence of amyloid fibril deposition in the RV, which is a defining characteristic of CA. As a result, an unusually thickened RV wall can serve as a potential clue to the coexistence of both conditions. Additionally, markers of RV systolic function, including TAPSE, tricuspid annular S’ velocity [61], and the RV strain, tend to show significantly greater impairment in patients with both AS and CA than in those with AS alone [63].

Recent advancements in advanced echocardiographic techniques, particularly myocardial work (MW) indices, have significantly enhanced diagnostic precision and clinical decision-making in patients with CA and related hypertrophic phenotypes. Global myocardial work indices, including the global work index (GWI), global constructive work (GCW), global wasted work (GWW), and global work efficiency (GWE), have emerged as powerful adjunctive tools for traditional echocardiographic parameters such as GLS. Recent comparative studies, such as the one in ref. [64] have demonstrated that MW analysis can effectively differentiate ATTR-CA from nonobstructive HCM, even in patients with preserved LVEF. Specifically, GWI and GCW were markedly impaired in ATTR-CA compared to HCM and hypertensive controls, underscoring their diagnostic potential. Notably, GWI has shown superior discriminative ability over GLS alone in distinguishing ATTR-CA from HCM in preserved LVEF scenarios, indicating its potential utility in clinical practice to improve early diagnosis and management strategies for patients presenting with overlapping hypertrophic cardiac conditions [64].

### 7.2. Cardiac Magnetic Resonance Imaging

CMR is a highly effective imaging technique for evaluating heart function and tissue properties. It provides detailed images with strong contrast between blood and tissue, aiding in the diagnosis of cardiomyopathies. Late gadolinium enhancement (LGE) detects cardiac fibrosis, while advanced parametric mapping (T1, T2, and extracellular volume (ECV) mapping) enables precise tissue characterization by measuring absolute numerical values. Moreover, the use of LGE and parametric T1 mapping can be used to evaluate extracellular volume [64].

In patients with AS, the CMR-evaluated extent of myocardial fibrosis is associated with the prognosis and recovery of LV systolic performance following aortic valve replacement [65].

In CA, CMR is an invaluable tool for providing important information. A hallmark feature of CA on CMR is the presence of diffuse subendocardial or transmural LGE, which results from amyloid infiltration altering the myocardial ECV. Unlike other cardiomyopathies where LGE follows coronary artery territories, the pattern of enhancement in CA is characteristically non-ischemic and involves both ventricles symmetrically. The most common and specific LGE pattern in CA is global subendocardial enhancement, although some patients may progress to a more transmural pattern as amyloid deposition increases. This finding is a key differentiator from HCM, where LGE typically appears in patchy, segmental, or mid-wall distributions [66]. Another critical CMR feature in CA is abnormal gadolinium kinetics, where patients exhibit difficulty in myocardial nulling during inversion-recovery sequences. This phenomenon, often referred to as the “nulling artifact,” occurs due to the extensive expansion of the interstitial space by amyloid fibrils, leading to rapid gadolinium washout from normal myocardium but delayed retention within amyloid-laden regions. This finding is highly suggestive of CA and further aids in distinguishing it from other hypertrophic conditions such as AS [67,68].

Beyond LGE, CMR parametric mapping techniques, particularly T1 and ECV mapping, have revolutionized the assessment of myocardial amyloid burden. In CA, native T1 relaxation times are significantly elevated due to amyloid infiltration, even before overt LGE appears. ECV mapping further enhances diagnostic precision by quantifying the proportion of the myocardium occupied by amyloid deposits, providing an objective measure of disease severity. Studies have shown that higher ECV values correlate with worse functional capacity, adverse outcomes, and higher mortality in CA patients [69,70].

Moreover, myocardial infiltration to the extent that it appears in the atria and to the right ventricle is not the case in AS.

Overall, the main distinguishing features of AS for CA, which can be used to detect the coexistence of the two entities, are present in the table (Table 1).

### 7.3. Cardiac Computed Tomography

CCT has become an invaluable imaging tool in the assessment of AS, particularly for evaluating aortic valve calcification and myocardial tissue characteristics. While echocardiography and CMR are the primary imaging modalities used to assess AS and CA, CCT provides complementary information that can aid in distinguishing isolated AS from the coexistence of AS and CA.

A key advantage of CCT in AS evaluation is its ability to quantify AVCS using non-contrast calcium scoring. In patients with severe AS, AVCS is typically extensive, with sex-specific AVCS thresholds established to define severe stenosis (≥3000 Agatston units in men and ≥1600 in women) [71]. However, in AS with coexisting CA, valve calcification may appear disproportionately low relative to the severity of stenosis measured by echocardiography or invasive hemodynamics. This discrepancy can serve as an important clue to underlying amyloid infiltration, as CA-related restrictive cardiomyopathy can exacerbate gradients across the valve despite relatively less calcification [72].

Beyond valve assessment, CCT provides detailed myocardial tissue characterization, particularly through late iodine enhancement (LIE) imaging using contrast-enhanced CT. Analogous to LGE on CMR, LIE in CCT can identify extracellular matrix expansion, which is a hallmark of amyloid deposition. Patients with CA often exhibit diffuse myocardial iodine retention, reflecting the amyloid-related expansion of the ECV. In contrast, myocardial fibrosis in isolated AS typically appears as patchy or mid-wall enhancement, which is a feature associated with chronic pressure overload. The ability to distinguish between global versus regional myocardial iodine enhancement provides an important diagnostic tool for recognizing amyloid involvement [73].

Another critical application of CCT in differentiating AS from AS + CA is ECV quantification through dual-energy CT (DECT) or equilibrium contrast-enhanced CT. ECV mapping with DECT provides a non-invasive means to measure the myocardial interstitial expansion characteristic of CA. Studies have shown that CA patients demonstrate markedly elevated ECV values (>40%), which correlate with the extent of amyloid infiltration. In contrast, patients with isolated AS may have mildly increased ECV (~30%), reflecting interstitial fibrosis rather than amyloid deposition. This distinction is particularly important in cases where CMR is contraindicated due to renal dysfunction, as CT-based ECV mapping offers an alternative method for tissue characterization [74,75].

Additionally, CCT enables the precise measurement of RV wall thickness, which is frequently increased in CA but not typically seen in isolated AS. The presence of an abnormally thickened RV (>6 mm on CT) should raise suspicion for coexisting CA.

CCT is instrumental in assessing RV wall thickness, which is often increased in CA but not typically in isolated AS. A study comparing CA and HCM patients found that CA patients had significantly thicker RV walls, averaging 7.8 ± 2.1 mm compared to 5.9 ± 1.3 mm in HCM patients. This suggests that an abnormally thickened RV wall (>6 mm) should raise suspicion for coexisting CA [76]. In contrast, isolated AS primarily affects the LV, with RV wall thickness typically remaining within normal limits. The normal thickness of the RV-free wall is less than 4 mm during end-diastolic pressure [76]. Therefore, the detection of increased RV wall thickness on CCT is a valuable diagnostic indicator, prompting further evaluation for potential CA in patients with AS.

### 7.4. Scintigraphy

BS with technetium−99m (99mTc)-radiolabeled bone-seeking tracers is the definitive and most accurate non-invasive method for diagnosing ATTR-CA. The most validated tracers are 99mTc-pyrophosphate (PYP), 3,3-diphosphono−1,2-propanodicarboxylic acid (DPD), and hydroxymethylene diphosphonate (HMDP).

A landmark study by Gillmore et al. involving 1217 patients with suspected amyloidosis demonstrated that the moderate-to-severe myocardial uptake of 99mTc-PYP/DPD/HMDP (Perugini grade 2–3) on BS exhibits high sensitivity (90%) and specificity (97%) for ATTR-CA. When combined with the absence of detectable monoclonal proteins, both specificity and positive predictive values reach 100%, reinforcing the diagnostic accuracy of this imaging modality [77].

The high sensitivity and specificity of BS enable the early detection of cardiac TTR amyloid deposition, even in asymptomatic individuals, preceding the onset of HF symptoms or structural abnormalities detectable by echocardiography or CMR [78]. This makes BS particularly valuable for screening high-risk patients, such as those with ATTR-AS, where studies have reported an ATTR-CA of 8–13% [3,4,5,41,55].

More specifically, Nitsche et al. screened 191 AS patients scheduled for TAVR using 99mTc-DPD scintigraphy and found an 8.4% prevalence of ATTR-CA [5]. This method exhibited superior diagnostic value compared to GLS by echocardiography, though it did not significantly differ from the voltage/mass ratio and stroke volume index [5].

Moreover, Rosenblum et al. examined AS patients undergoing TAVR with 99mTc-PYP and identified a 13% prevalence of ATTR-CA [55]. These patients demonstrated an increased rate of HF hospitalization following TAVR, highlighting the clinical implications of the underlying cardiomyopathy [55].

Similarly, Scully et al. evaluated 200 patients undergoing TAVR and used 99mTc-DPD scintigraphy to detect a 13% prevalence of ATTR-CA, which is consistent with the findings of Rosenblum et al. [41]. Notably, TAVI significantly improved clinical outcomes for all patients, regardless of ATTR-CA presence [41]. The prevalence of ATTR-CA detected by scintigraphy in AS patients (8–13%) in the aforementioned studies was lower than the 25% reported in autopsy studies of individuals over 80 years of age, but this value is more realistic and indicates the most accurate diagnosis of amyloid cardiomyopathy compared to TTR deposition alone [5,15,41,55].

## 8. The Role of Artificial Intelligence in Diagnosis and Early Identification

Artificial intelligence (AI) has emerged as a promising tool for the early detection and screening of ATTR-CA in patients with AS. Recent studies have demonstrated the potential of AI models to identify this underdiagnosed entity using widely available diagnostic modalities such as ECGs and echocardiograms. Goto et al. developed an AI-based pipeline capable of achieving high diagnostic accuracy for CA, with C-statistics of 0.85–0.91 for ECGs and 0.89–1.00 for echocardiography across multiple cohorts [79]. This model demonstrated robust performance in detecting CA, including ATTR-CM, which represented more than half of the cases. Importantly, the pipeline was not restricted to AS patients, highlighting its applicability in diverse settings. Furthermore, pre-screening with ECG models improved the positive predictive value (PPV) of echocardiography models from 33% to 74–77%, highlighting the potential of a combined AI approach to be integrated into clinical workflows, augmenting the diagnostic accuracy in detecting such low-prevalence conditions.

In addition to ECG and echocardiography, AI has been applied to other imaging modalities to detect CA, specifically in AS patients. A multi-modality AI approach, using clinical, laboratory, ECG, echocardiography, and CT data, achieved an area under the curve (AUC) of 0.84 for ATTR-CA detection in a population with severe AS, where ATTR-CA represented 10.3% of the cases [80]. This study also demonstrated that AI models could provide prognostic information, with the multi-modality model effectively discriminating between low- and high-risk patients for all-cause mortality over an average follow-up period of 13 months. Similarly, another study utilized an AI algorithm to analyze pre-TAVR ECGs, identifying 24.4% of patients as high-risk for CA. These patients had significantly worse outcomes, including increased all-cause mortality (adjusted hazard ratio [aHR]: 1.40, 95% CIs: 1.01–1.96, *p*: 0.046), major adverse cardiovascular events (aHR: 1.36, 95% CIs: 1.01–1.82, *p*: 0.041) and HF hospitalizations (aHR: 1.58, 95%CIs: 1.13–2.20, *p*: 0.008), underscoring the prognostic value of AI in risk stratification [81].

The integration of AI into clinical practice offers a cost-effective and non-invasive strategy for ATTR-CA screening, particularly in high-risk populations such as AS patients, where the “naked eye” distinction between the imaging phenotypes of the two conditions is often challenging. Future studies should focus on the external validation of these models and their implementation in diverse clinical settings to ensure generalizability and real-world applicability [82].

## 9. Therapeutic Approaches in Patients with Coexisting Cardiac Amyloidosis and Aortic Stenosis

The coexistence of CA and AS is associated with a worse prognosis compared to either condition alone. Multiple studies have highlighted the increased mortality risk in patients with both diseases, emphasizing the need for tailored therapeutic strategies.

A meta-analysis involving 4243 patients from 21 studies demonstrated that individuals with both AS and CA had a significantly higher mortality rate than those with AS or CA alone [31,44]. Additionally, an analysis of nine studies that included 178 AS patients with CA and 1220 patients with AS alone found that the presence of CA was associated with an increased mortality risk (OR: 2.25, 95% CI: 1.23–3.94, *p* = 0.004), with moderate heterogeneity among the studies (I^2^ = 43%, *p* = 0.082) [39].

In patients with cardiac amyloidosis, aortic stenosis, or a combination of both, the presence of HF necessitates the initiation of guideline-directed medical therapy whenever possible. Standard HF treatments, including beta-blockers, renin–angiotensin system inhibitors, and mineralocorticoid receptor antagonists, play a crucial role in symptom management and improving clinical outcomes. However, the therapeutic landscape is evolving, and the role of sodium–glucose cotransporter 2 (SGLT2) inhibitors appears to extend beyond conventional HF management, offering potential benefits even in the absence of overt HF. Recent evidence suggests that SGLT2 inhibitors may be beneficial in both ATTR-CM and AS. Although ATTR-CM patients have been excluded from major randomized trials of SGLT2 inhibitors, real-world data indicate that their use is well tolerated and associated with favorable outcomes. In a large observational study, SGLT2 inhibitor therapy in ATTR-CM was linked to improved heart failure symptoms, preserved renal function, and a reduced need for diuretics while also significantly lowering the risk of heart failure hospitalization and cardiovascular mortality [83]. Similarly, in patients with severe AS, reduced left ventricular ejection fraction, and extra-valvular cardiac damage undergoing transcatheter aortic valve implantation (TAVI), the use of SGLT2 inhibitors was associated with improved left ventricular recovery and reduced adverse cardiovascular events. Notably, SGLT2 inhibitor users had a lower incidence of all-cause mortality and heart failure hospitalization after two years, particularly among those with a severely impaired baseline ejection fraction [84]. These findings suggest that SGLT2 inhibitors may provide additional therapeutic benefits beyond glycemic control, warranting further investigation in dedicated clinical trials.

Initial studies suggested that TAVR outcomes were poorer in patients with CA; however, these studies were poorly designed. More recent well-structured studies have shown improved outcomes in patients with coexisting AS and CA, with results comparable to the general AS population.

Interestingly, the prevalence of ATTR-CA in AS patients at expert centers has declined over the years, decreasing from 16% to 5%. This trend likely reflects the increased awareness of ATTR-CA and the expansion of TAVR to lower-risk populations [85].

### 9.1. Transcatheter Aortic Valve Replacement in Cardiac Amyloidosis and Aortic Stenosis

TAVR has been shown to improve survival in patients with coexisting CA and AS compared to medical therapy alone [1]. A meta-analysis confirmed that patient survival following TAVR was similar between those with CA-AS and those with AS alone [31,78]. Additionally, outcomes in patients undergoing TAVR with CA-AS were comparable to those with AS alone, with no significant differences for stroke, vascular complications, life-threatening bleeding, acute kidney injury, or 30-day mortality [31,86]. However, there was a trend toward a higher risk of permanent pacemaker implantation in the CA-AS group [31,86]. Moreover, a meta-analysis including seven observational studies found that TAVR significantly reduced mortality in CA-AS patients compared to medical therapy [86]. Another meta-analysis including 83 patients (45 undergoing TAVR, 38 managed conservatively) found a significantly lower 12-month all-cause mortality risk in the TAVR group compared to medical therapy [87].

A large retrospective analysis of the National Inpatient Sample (NIS) database assessed 304,710 hospitalizations for TAVR between 2016 and 2020, identifying 410 patients (0.14%) with ATTR-CA. After adjusting for patient characteristics, ATTR-CA was not associated with a significant increase in in-hospital mortality [88]. There were also no significant differences in the rates of heart block, permanent pacemaker insertion, stroke, acute kidney injury, major bleeding, the need for blood products, or vascular injuries between the ATTR-CA and AS groups [88].

Although TAVR leads to symptomatic improvement in both CA-AS and AS patients, those with CA-AS tend to remain more symptomatic. A study of 120 patients undergoing TAVR found that at 12 months, AS-ATTR patients had significantly higher residual NT-proBNP levels and no significant regression in LV mass, whereas AS patients exhibited significant LV mass regression [89]. Furthermore, echocardiographic remodeling patterns differ between the two groups, with AS-ATTR patients developing an apical sparing pattern after TAVR [89].

Regarding long-term prognosis and valve durability at a median follow-up of 1.7 years, there is a trend toward higher 1-year mortality in AS-CA patients compared to lone AS, but after adjustment for baseline differences, coexisting CA is not an independent predictor of adverse outcomes [5,47]. Additionally, concerns have been raised that amyloid infiltration into bioprosthetic valve leaflets may contribute to accelerated structural valve deterioration, though further research is needed to determine if CA-AS patients experience shorter valve durability after TAVR [47].

While TAVR has been shown to improve survival and reduce mortality in patients with coexisting CA and AS, further investigation into post-procedural outcomes and long-term follow-up is necessary to refine treatment strategies. While immediate procedural success is often achieved, patients with CA-AS may continue to experience significant challenges due to underlying restrictive cardiomyopathy, such as persistent heart failure symptoms, impaired diastolic function, and a higher likelihood of requiring pacemaker implantation. Long-term data on survival, heart failure progression, and the impact of TAVR on overall quality of life in this cohort remain limited. Additionally, the potential for accelerated bioprosthetic valve deterioration due to amyloid infiltration warrants further exploration to determine the durability of TAVR in CA-AS patients. Further studies focusing on long-term outcomes and the effect of disease-modifying therapies would be invaluable in optimizing post-TAVR management and ensuring the best outcomes for these patients.

### 9.2. Comparing TAVR and SAVR in Patients with Aortic Stenosis and Cardiac Amyloidosis

Studies comparing TAVR and SAVR in AS-CA patients show mixed results. Early small-cohort studies (*n* < 30) suggested a TAVR advantage [55,56], while newer data provide further insights. Most AS-CA patients are ineligible for SAVR due to age and comorbidities, making TAVR the preferred choice. In the study by Nitsche et al., 82% received TAVR, 16% were medically treated, and only 3% underwent SAVR [1]. Several studies indicate that TAVR lowers mortality compared to medical therapy, with no significant difference between TAVR and SAVR (*p* = 0.217) [39].

SAVR is associated with higher procedural risks in CA patients. A large study using the National Inpatient Sample found that SAVR had higher rates of acute kidney injury, myocardial infarction, and major bleeding compared to TAVR. Mortality was also higher in SAVR (6% vs. 4%, *p* < 0.01), with longer hospital stays (14 vs. 9 days) [90,91]. These findings suggest that TAVR is safer and more efficient, particularly for frail elderly patients. Additionally, severe diastolic dysfunction in CA complicates SAVR due to difficulties in weaning from cardiopulmonary bypass, whereas TAVR remains unaffected [90].

Recent studies found no significant difference in mortality or major cardiovascular events between TAVR and SAVR [92]. However, in-hospital mortality was lower for TAVR (4.4%) vs. SAVR (11.9%) [93], with fewer complications such as acute kidney injury and sepsis. While further research is needed, current evidence suggests that TAVR offers a superior safety and efficacy profile over SAVR, with treatment decisions individualized based on patient characteristics (Table 2).

#### 9.2.1. Transthyretin Stabilizers

Tafamidis is an orally bioavailable benzoxazole derivative that stabilizes the TTR tetramer by binding to T4-binding sites without exhibiting nonsteroidal anti-inflammatory activity [95]. Clinical benefits become significant after 18 months, with more pronounced effects when administered early in the course of CA, particularly in patients with NYHA functional class I and II HF [56]. The trial demonstrated a 13.4% absolute reduction in all-cause mortality in the pooled tafamidis cohort compared with the placebo (29.5% vs. 42.9%) and a 32% lower risk of cardiovascular hospitalizations in NYHA class I or II patients over 30 months. However, patients with NYHA class III symptoms experienced higher cardiovascular hospitalization rates with tafamidis than with a placebo, emphasizing the importance of early diagnosis [96]. TTR stabilization therapy with tafamidis should be initiated as soon as ATTR-CA is diagnosed, independent of any aortic valve replacement (SAVR or TAVR) [56]. In patients with AS-CA, tafamidis was used in a minority (14.9%, 7 of 47) post-AVR and was not associated with a mortality difference (log-rank; *p* = 0.40) [5].

Another TTR stabilizer, diflunisal, is a nonsteroidal anti-inflammatory drug (NSAID) with TTR-stabilizing properties. It binds to T4-binding sites at the dimer–dimer interface of the TTR tetramer, preventing dissociation into amyloidogenic monomers and oligomers [95]. While diflunisal can cause adverse effects such as gastrointestinal hemorrhage, renal dysfunction, fluid retention, and hypertension, its dose for ATTR-CA treatment (250 mg twice daily) is lower than that used for anti-inflammatory purposes and is generally well tolerated.

Acoramidis (AG10) represents a newer approach to TTR stabilization, mimicking the structural stabilizing properties of the TTR variant *p*.T139M, which protects against familial amyloid polyneuropathy in heterozygous carriers of the disease-causing *p*.V50M variant [95,97]. Compared to tafamidis and diflunisal, acoramidis exhibits superior potency and selectivity, binding more selectively to serum TTR and providing enhanced stabilization [97]. Unlike tafamidis, it kinetically stabilizes both wild-type and variant *p*.V142I TTR tetramers. In a Phase 2 study, acoramidis achieved near-complete TTR stabilization (>90%) and increased serum TTR levels by >50% at 28 days in patients receiving 800 mg twice daily versus the placebo [97].

The ATTRIBUTE-CM Phase 3 trial (NCT03860935) enrolled 510 ATTR-CA patients randomized 2:1 and received treatment with either acoramidis (800 mg twice daily) or a placebo for 30 months, assessing changes in the 6 min walk test (6MWT) distance that patients achieved at 12 months and all-cause mortality and cardiovascular hospitalizations over 30 months [95]. A total of 632 patients were randomized, with the primary analysis favoring acoramidis over a placebo (*p* < 0.001). The win ratio was 1.8 (95% CI: 1.4–2.2), with 63.7% of pairwise comparisons favoring acoramidis. The incidence of adverse events was similar between the acoramidis and placebo groups (98.1% vs. 97.6%), with serious adverse events reported in 54.6% and 64.9% of patients, respectively [98].

Acoramidis treatment led to improved biventricular function at month 30 compared to the placebo. LVEF increased by 4.6% (mean baseline: 50.7%) in acoramidis recipients but declined by 8.2% in placebo recipients. Indexed LV stroke volume improved with acoramidis (median increase: 6.5 mL/m^2^) but declined in the placebo group (−3.0 mL/m^2^). RV function also improved, with the RV ejection fraction increasing by 1.8% in the acoramidis group but deteriorating by 9.6% in the placebo group [99].

In this study, extension to month 42 with continuous acoramidis treatment showed sustained benefits. The hazard ratio (HR) for all-cause mortality or first cardiovascular hospitalization was 0.57 (95% CI: 0.46–0.72; *p* < 0.0001), with HRs of 0.64 and 0.53 for all-cause mortality and first cardiovascular hospitalization alone, respectively [100]. Quality of life, assessed by the Kansas City Cardiomyopathy Questionnaire-Overall Summary (KCCQ-OS), was better preserved in continuous acoramidis recipients. No new safety concerns emerged in this long-term evaluation.

Currently, there are no dedicated clinical trials assessing the impact of disease-modifying therapies for transthyretin amyloidosis, such as tafamidis, acoramidis, or patisiran, on the progression of AS or outcomes following TAVR. While these therapies have demonstrated efficacy in stabilizing or reducing amyloid deposition in cardiac amyloidosis, their potential effects on AS progression remain unexplored. Given the increasing recognition of amyloid infiltration in patients with AS, future research should focus on investigating whether these agents could modify the natural history of AS, improve post-TAVR outcomes, or alter disease progression in this patient population.

#### 9.2.2. Transthyretin Knockdown/Silencing Therapies

Patisiran is a small interfering RNA (siRNA) formulated as a lipid nanoparticle to facilitate hepatic uptake, blocking the liver’s production of both normal and mutated TTR [56,101]. The APOLLO Phase 3 trial (NCT01960348) randomized 225 patients with ATTR familial amyloid polyneuropathy (NYHA functional class I-II) in a 2:1 ratio to receive patisiran (0.3 mg/kg every 3 weeks) or a placebo [38]. A sub-analysis in the CA subset showed reverse cardiac remodeling, increased cardiac output, and reduced N-terminal pro-brain natriuretic peptide levels but no significant reduction in mortality or rehospitalization [56,101]. Expanding on this, the APOLLO-B trial (NCT03997383) enrolled 300 patients with ATTR-CA (excluding NYHA class IV) in a 1:1 ratio to either patisiran or placebo [95]. After 12 months, patisiran treatment resulted in a smaller decline in the 6MWT distance (median difference: 14.69 m; *p* = 0.02) and an increase in the KCCQ-OS score (difference: 3.7 points; *p* = 0.04). However, significant benefits were not observed for secondary endpoints such as mortality and cardiovascular events [102]. A post hoc analysis of APOLLO-B revealed that outpatient worsening with HF was associated with increased mortality and cardiovascular events, while patisiran reduced the risk of outpatient worsening with HF (HR: 0.70; 95% CI: 0.51–0.96) over 24 months [103].

Another RNA-targeting therapy, inotersen, is an FDA-approved antisense oligonucleotide (ASO) designed to reduce the production of both normal and mutant TTR proteins. In the NEURO-TTR trial (NCT01737398), weekly subcutaneous injections of 300 mg of inotersen stabilized neuropathy and quality of life in ATTR patients, regardless of cardiac involvement [104]. However, concerns arose due to severe adverse events, including glomerulonephritis (3%), severe thrombocytopenia (3%), and one fatal intracranial hemorrhage. While the open-label extension (NCT02175004) demonstrated improved safety monitoring and reduced thrombocytopenia incidence [95,104], the emergence of longer-acting ASOs with better safety profiles led to a decline in active research on inotersen for ATTR-CA [95,104].

In contrast, vutrisiran, a second-generation siRNA therapy, was developed with improved hepatic targeting via the N-acetyl galactosamine (GalNAc) conjugation, which enhances liver uptake through the asialoglycoprotein receptor (ASGPR). A Phase 1 study demonstrated that vutrisiran reduced plasma TTR levels by 83% after 6 weeks, with sustained effects for up to 90 days. Further evaluating its efficacy, the HELIOS-B trial (NCT04153149) enrolled 600 ATTR-CA patients, randomized to receive vutrisiran (25 mg) or a placebo every 12 weeks for up to 36 months. The results demonstrated a significant reduction in the risk of death from any cause and recurrent cardiovascular events with vutrisiran compared to the placebo (hazard ratio: 0.72; 95% CI: 0.56–0.93; *p* = 0.01), with even more pronounced benefits in the monotherapy population (HR: 0.67; 95% CI: 0.49–0.93; *p* = 0.02) [105]. Additional findings indicated a lower risk of death from any cause throughout 42 months (HR: 0.65; *p* = 0.01), and patients receiving vutrisiran experienced less functional decline, as evidenced by a smaller reduction in the 6MWT distance (least-squares mean difference: 26.5 m; *p* < 0.001) and a more preserved KCCQ-OS score (least-squares mean difference: 5.8 points; *p* < 0.001). The safety profile was favorable, with similar adverse event rates in both the vutrisiran (99%) and placebo (98%) groups and serious adverse events occurring in 62% and 67% of patients, respectively [105].

Eplontersen, also known as ION-682884, represents a further evolution in ASO therapy for ATTR, particularly in cases involving cardiomyopathy (ATTR-CM). When engineered for selective liver uptake via GalNAc conjugation, eplontersen aims to preferentially reduce mutant TTR levels while preserving more normal TTR, which is a key improvement over inotersen. In Phase 1 studies, eplontersen achieved an 85.7% mean reduction in plasma TTR levels with a monthly subcutaneous dose of 45 mg, demonstrating a better safety profile than its predecessor [101]. Expanding on this, the NEURO-TTRansform study evaluated eplontersen in 144 adults with hATTR polyneuropathy, 49 (34%) of whom had cardiomyopathy. At 65 weeks, eplontersen treatment resulted in significant cardiac function improvements, including a 4.3% increase in LVEF (*p* = 0.049) and a 10.64 mL increase in stroke volume (*p* = 0.002) compared to the historical placebo group. Other echocardiographic parameters remained stable, suggesting the potential for eplontersen to both maintain and enhance cardiac function in ATTR-CM [101].

To further establish its role, the ongoing Phase 3 CARDIO-TTRansform trial (NCT04136171) is evaluating eplontersen in 750 patients with ATTR-CA. Participants are randomized to receive either eplontersen or a placebo every 4 weeks, with the final results expected in 2026. Preliminary data, including findings from NEURO-TTRansform, suggest that eplontersen may offer meaningful benefits in preserving cardiac function and slowing disease progression, making it a promising candidate for the future treatment of ATTR-CM [101,106].

#### 9.2.3. Emerging Agents for Degradation/Extraction

PRX-004 is an intravenous monoclonal antibody that targets the misfolded TTR amyloid, aiming to clear amyloid deposits from the myocardium while preserving native TTR. A Phase 1 study (NCT03336580) demonstrated that PRX-004 was safe and well tolerated, with no serious adverse events reported [101]. Building on the concept of targeting misfolded TTR, NI006, a recombinant human monoclonal antibody, has also been developed for both wild-type and hereditary ATTR. In a Phase 1 study (NCT04360434), NI006 showed promising results in reducing cardiac amyloid load, as indicated by improvements in cardiac tracer uptake on the scintigraphy and extracellular volume on CMR. Additionally, the absence of drug-related serious adverse events highlights its potential as a therapeutic option for ATTR cardiomyopathy [107].

Dezamizumab, an anti-SAP antibody, was studied for its ability to clear amyloid deposits in a Phase 1 study, showing reductions in liver stiffness and renal amyloid load. However, a Phase 2 study (NCT03044353) failed to show improvements in cardiac amyloid burden in ATTR-CA, and this drug is no longer under investigation for cardiac use due to an unfavorable risk–benefit profile [108].

**Figure 3:**
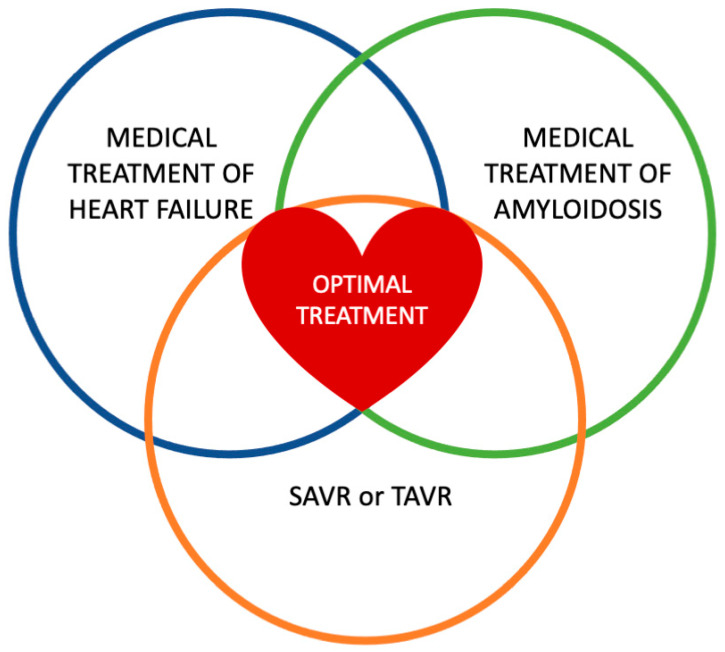
This Venn diagram illustrates the three key components of aortic stenosis with concomitant cardiac amyloidosis treatment, represented by overlapping circles. Each circle highlights a distinct aspect of management: the medical treatment of heart failure focuses on symptom relief and hemodynamic optimization, considering the unique challenges posed by amyloid−related cardiac dysfunction. The medical treatment of amyloidosis encompasses disease−modifying therapies, including anti−amyloid agents, aimed at slowing or halting disease progression. SAVR or TAVR for aortic stenosis represents the procedural options for valve replacement, tailored to individual patient risk and amyloid−related cardiac involvement. The central overlapping area signifies the intersection between these treatment strategies, emphasizing the need for an integrated, multidisciplinary approach to optimize patient outcomes.

These emerging treatment options have been evaluated exclusively in patients with CA and not in those with AS and CA. Future studies are needed to assess the efficacy and safety of these drugs in this specific population and to compare their outcomes with TAVR and SAVR. Additionally, investigations should explore the potential benefits of combining these medical therapies with interventional approaches to optimize patient management (Figure 3).

## 10. Conclusions

The coexistence of AS and ATTR-CA presents significant diagnostic and therapeutic challenges. Advances in imaging and biomarkers have improved detection, yet distinguishing AS-CA from isolated AS remains difficult. TAVR is a viable treatment, though patients with AS-CA experience higher HF risks post-procedure. Emerging therapies, including tafamidis and gene-silencing agents, offer promise in slowing disease progression. Routine screening and a multimodal approach are essential for early diagnosis and optimized management. Future research should refine diagnostic tools and treatment strategies.

## Figures and Tables

**Table 1:** Distinctive CMR features of AS and AS-CA.

Characteristics	AS	AS-CA
LGE pattern	Patchy/mid-wall and localized fibrosis	Global subendocardial/transmural
T1 mapping	Mildly elevated	Markedly elevated
ECV fraction	Mildly elevated	Highly elevated (>40–50%)
Nulling pattern	Normal nulling	Abnormal nulling (blood nulls before myocardium)
Atrial size	Left atrial enlargement	Bi-atrial enlargement
Right ventricular involvement	Uncommon	Frequent

Abbreviations: CMR, cardiac magnetic resonance; AS, aortic valve stenosis; AS-CA, aortic valve stenosis with cardiac amyloidosis.

**Table 2:** Comparison of TAVR and SAVR in patients with AS-CA: key studies and outcomes.

Study	Design and Population	Sample Size	Key Findings
Myasoedova et al. (2022) [39]	Systematic review and meta-analysis	10 studies (330 AS-CA patients)	SAVR and TAVR had a similar overall mortality risk but were lower than medication-treated only patients
Khan et al. (2022) [91]	Retrospective cohort	208,540 patients—TAVR (18,745, 8.9%) and SAVR (189,795, 91.01%)	SAVR was associated with higher rates of acute kidney injury, acute myocardial infarction, and major bleeding compared to TAVR. The TAVR group had a higher incidence of stroke, vascular complications, and permanent pacemaker implantation. In the SAVR group, mortality was higher in 2011 and 2014 but was not statistically different in 2010, 2012, and 2013. The length of stay was longer, and the cost was higher in the SAVR group annually compared to the TAVR group
Mir et al. (2024) [93]	Retrospective study—national cohort study	4820 patients AS-CA (464 intervention: 251 TAVR, 213 SAVR)	AS patients with amyloidosis and in-hospital mortality were significantly lower for patients undergoing TAVR (4.4%) compared to SAVR (11.9%). Complications such as acute kidney injury, sepsis, and cardiogenic shock were more frequent in the SAVR group Acute heart failure was higher among patients who had TAVR All conduction blocks and cases of ischemic stroke were similar between the two groups (*p* = 0.09 and *p* = 0.1)
Masri et al. (2025) [92]	Retrospective analysis	1239 patients	Patients with AS-CA who underwent TAVR showed no significant difference in mortality or major cardiovascular events compared to those who underwent SAVRTAVR patients were older and had more comorbidities, including HF and chronic kidney disease, yet outcomes were similar between the two procedures
Ahmad et al. (2025) [94]	Systematic review and meta-analysis	15 studies (6704 patients)	TAVR was associated with lower all-cause mortality compared to both medical therapy (RR = 0.50, 95% CI 0.29–0.89, *p* = 0.02) and SAVR (RR = 0.41, 95% CI 0.22–0.78, *p* = 0.007)

Abbreviations: AS, aortic stenosis; AS-CA, aortic stenosis with cardiac amyloidosis; CI, confidence interval; HF, heart failure; RR, relative risk; SAVR, surgical aortic valve replacement; TAVR, transcatheter aortic valve replacement.9.3. Transthyretin Stabilizers and Knockdown Therapies

## Abbreviations

The following abbreviations are used in this manuscript:

AI	Artificial Intelligence
AL-CA	Light-Chain Cardiac Amyloidosis
AS	Aortic Stenosis
AF	Atrial Fibrillation
AS-CA	Aortic Stenosis and Cardiac Amyloidosis
ASGPR	Asialoglycoprotein Receptor
ASO	Antisense Oligonucleotide
ATTR	Transthyretin Amyloidosis
ATTR-CA	Transthyretin Amyloidosis Cardiac Amyloidosis
AUC	Area Under the Curve
AVCS	Aortic Valve Calcium Scores
CA	Cardiac Amyloidosis
CMR	Cardiovascular Magnetic Resonance
CCT	Cardiac Computed Tomography
CTS	Carpal Tunnel Syndrome
DECT	Dual-Energy Computed Tomography
DPD	Technetium−99m 3,3-Diphosphono−1,2-Propanodicarboxylic Acid Bone Scintigraphy
ECG	Electrocardiogram
ECV	Extracellular Volume
EF	Ejection Fraction
EMR	Electronic Medical Record
GalNAc	N-acetyl Galactosamine
GLS	Global Longitudinal Strain
HCM	Hypertrophic Cardiomyopathies
HF	Heart Failure
HFpEF	Heart Failure with Preserved Ejection Fraction
HMDP	Hydroxymethylene Diphosphonate
hATTR	Hereditary Transthyretin Amyloidosis
hsTnT	High Sensitive Troponin T
KCCQ-OS	Kansas City Cardiomyopathy Questionnaire-Overall Summary
LA	Left Atrium
LFLG-AS	Low-Flow, Low-Gradient Aortic Stenosis
LGE	Late Gadolinium Enhancement
LIE	Late Iodine Enhancement
LV	Left Ventricular
LVH	Left Ventricular Hypertrophy
MCF	Myocardial Contraction Fraction
NIS	National Inpatient Sample
NSAID	Nonsteroidal Anti-inflammatory Drug
NT-proBNP	B-type Natriuretic Peptide
PSM	Propensity-Score Matching
PYP	Pyrophosphate
RV	Right Ventricle
RVHF	Right Ventricular Heart Failure
SAVR	Surgical Aortic Valve Replacement
siRNA	Small Interfering RNA
STE	Speckle-Tracking Echocardiography
TAPSE	Tricuspid Annular Plane Systolic Excursion
TAVR	Transcatheter Aortic Valve Replacement
TAVI	Transcatheter Aortic Valve Implantation
TDI	Tissue Doppler Imaging
TTE	Transthoracic Echocardiography
TTR	Transthyretin
VMR	Voltage/Mass Ratio
wtATTR	Wild-Type Transthyretin Amyloidosis
